# Susceptibility to tobacco use and associated factors among youth in five central and eastern European countries

**DOI:** 10.1186/s12889-022-12493-6

**Published:** 2022-01-11

**Authors:** Kinga Polanska, Malgorzata Znyk, Dorota Kaleta

**Affiliations:** Department of Hygiene and Epidemiology, Medical University of Lodz, 7/9 Zeligowskiego Str, 90-647 Lodz, Poland

**Keywords:** GYTS, Susceptibility, Tobacco use, Cigarettes, Youth, Central and Eastern Europe

## Abstract

**Background:**

Tobacco use among young people still remains a major public health problem. Thus, the aim of this study was to perform a cross-country comparison for the factors associated with susceptibility to tobacco use among youth from five central and eastern European countries.

**Methods:**

The data used in the current analysis, focusing on youth (aged 11–17 years), who have never tried or experimented with cigarette smoking, was available from the recent Global Youth Tobacco Survey (Czech Republic (2016), *n* = 1997; Slovakia (2016), *n* = 1998; Slovenia (2017), *n* = 1765; Romania (2017), *n* = 3718; Lithuania (2018), *n* = 1305). Simple, multiple logistic regression analyses and random-effect meta-analysis were conducted to identify factors associated with tobacco use susceptibility as the lack of a firm commitment not to smoke.

**Results:**

Nearly a quarter of the students were susceptible to tobacco use in 4 of 5 countries. The following factors were identified, consistently across countries, as correlates of tobacco use susceptibility: exposure to passive smoking in public places (AOR from 1.3; *p* = 0.05 in Slovakia to 1.6; *p* < 0.01 in Czech Republic and Romania), peers smoking status (AOR from 1.8 *p* < 0.01 in Slovakia to 2.5; *p* < 0.01 Lithuania), opinion that smoking helped people feel more comfortable at celebrations (AOR from 1.3; *p* = 0.01 in Czech Republic to 1.9; *p* < 0.01 in Lithuania), noticing people using tobacco in mass media (AOR 1.5; *p* < 0.01 in Slovenia and 1.6; *p* < 0.01 in Lithuania), lack of knowledge on harmful effects of passive smoking (AOR 1.8; *p* < 0.01 in Slovakia and 2.4; *p* < 0.01 in Slovenia), lack of antismoking education provided by school (AOR 1.3; *p* < 0.05 in Czech Republic, Slovakia and Slovenia; 1.9; *p* < 0.01 in Lithuania), and family (AOR 1.5; *p* < 0.01 in Slovenia and Romania). Moreover those who believed that smoking makes young people look less attractive (AOR from 0.5; *p* < 0.01 in Romania to 0.7; *p* = 0.05 in Lithuania) and that people who smoke have less friends (AOR 0.7; *p* ≤ 0.06) turned out to be less susceptible to tobacco use initiation. In Czech Republic and Slovenia significantly higher susceptibility to tobacco use was observed among females as compared to males (AOR 1.4; *p* < 0.01), whereas in Romania opposite pattern, although not significant, was observed (*p* = 0.3). Having more money available for own expenses, positively correlated with smoking suitability in all countries (AOR > 1.5; *p* < 0.01) except Lithuania where youth with more money available tend to be less susceptible to tobacco use (*p* > 0.05). Youth who share the opinion that people who smoke have more friends were more susceptible to smoking in Romania (AOR 1.4; *p* = 0.04) but tend to be less susceptible in other countries. Exposure to advertisements at points of sale was significant correlate of tobacco use susceptibility in Slovakia and Slovenia (AOR 1.4 and 1.5 respectively; *p* < 0.05), with moderate heterogeneity between the countries.

**Conclusions:**

A high proportion of youth from central and eastern European countries was susceptible to tobacco use. Social factors, and those related to educational and policy issues as well as to attitudes regarding tobacco use were strongly, and consistently across countries, correlated with tobacco use susceptibility. Slight differences in susceptibility to tobacco use between the countries were related to: sex, money available for own expenses, exposure to advertisements at points of sale and opinion that people who smoke have more friends. These factors should be considered when designing and implementing anti-tobacco activities among young people.

**Supplementary Information:**

The online version contains supplementary material available at 10.1186/s12889-022-12493-6.

## Background

Tobacco use among young people remains a major public health problem worldwide. Adolescents are at risk of experimenting with various tobacco and nicotine products [1]. Data shows that 88% of current adult tobacco smokers start smoking before the age of 18 and that about one fifth of adolescents around the world smoke tobacco with multiple effects on their health (increased mortality and morbidity from non-communicable diseases (NCDs), reduced life expectancy), economy and social, peer and family integrity [2–5]. Moreover, existing studies indicate that individuals who started smoking at a younger age usually smoke more cigarettes per day, are at higher risk of nicotine addiction, are less likely to try to quit smoking or if they do so, they have less chance to quit comparing to those who start smoking as adults [6–8]. They are also prone to coexisting risky behaviours such as alcohol or illicit drug use [9].

According to the recent WHO Report on the Global Tobacco Epidemic [10] there were 1.07 billion smokers aged 15 years and above, worldwide. In addition, 24 million children aged 13–15 smoked globally. In Europe, adult and adolescent smoking was among the highest in WHO regions, with 28 and 17%, respectively. Tobacco consumption among young people is growing, in some countries, such as: Lithuania, Latvia and Czech Republic, reaching almost similar rates as in adults [11]. The recent Global Youth Tobacco Survey (GYTS) conducted in central and eastern European countries indicates the following percentages of current smokers (youth who declared smoking within the last 30 days) among 13–15 year old adolescents: Czech Republic: 15%, Slovakia 17%, Lithuania 17%, Romania 9% and Slovenia 6% [12].

Preventing minors from starting tobacco use is more effective and it costs less than helping users to quit. An important public health challenge is, therefore, to identify factors that influence propensity to use tobacco among young people and predict their likelihood of such a behavior in the future [8]. Knowing them is crucial for development of effective preventive measures to reduce tobacco use among young people, and thus, reduce the frequency of using that products. According to its definition, susceptibility to smoking is the lack of a firm commitment not to smoke and it is a strong predictor of regular smoking and addiction [13]. Studies indicate that youth who are susceptible to tobacco use are more likely to experiment with tobacco and to become regular smokers compared to those non-susceptible ones. Moreover, susceptibility to tobacco use has been shown to be modifiable through interventions (which can either prevent youth from becoming susceptible to tobacco use or prevent susceptible adolescents from progressing to regular smokers) [14]. Various factors are indicated as correlates of tobacco use susceptibility among youth, including: individual characteristics (e.g. age, gender), social environment and social contexts (e.g. family, friends, school) [8, 15–21]. As an example, the analysis based on GYTS (conducted in 168 countries between 1999 and 2008) indicated that exposure to parental or peer smoking, antismoking media messages, secondhand smoke inside or outside home and tobacco industry promotion were associated with increased smoking susceptibility [18]. Moreover, support for smoke-free policies and school antismoking education were associated with decreased susceptibility to smoking among females. However, such factors can be region or country specific and can change over the years as a response to relevant tobacco policies, social and cultural norms, tobacco industry influence as well as preventive activities.

GYTS, which has been conducted recently in central and eastern European countries (Czech Republic, Slovakia, Lithuania, Slovenia and Romania), creates a unique opportunity to evaluate or update the factors that predispose young people to be susceptible to tobacco use, which in turn can be useful for development and implementation of effective tobacco control strategies in these countries [12]. As it was mentioned above in 3 out of 5 countries smoking prevalence among youth was relatively high [12]. Moreover, although these countries represent cultural similarities some differences in compliance with WHO tobacco control measures were observed. According to WHO assessments in Romania, all public places and in Czech Republic, Slovakia, Lithuania three up to five public places were indicated as completely smoke-free [10, 22]. In Slovenia the data was not reported; however, this country had one or more public places where designated smoking rooms were allowed. There was no national campaign conducted close to the survey period in Czech Republic, Slovakia and Lithuania. National campaign with one to four appropriate characteristics was conducted at that time in Slovenia and in the preceding years in Romania. In all the analyzed countries health warnings with appropriate characteristics were in place. Finally, only in Slovenia there was a ban on all forms of direct and indirect advertising, whereas in the other countries there was a ban on national television, radio and print media as well as on some but not all other forms of direct and/or indirect advertising.

Considering all the above, the aim of the study was to perform a cross-country comparison for the factors associated with susceptibility to tobacco use among youth from five central and eastern European countries.

## Methods

### Study design and population

The data used in the current analysis is from GYTS, a part of Global Tobacco Surveillance System (GTSS), which was developed to track tobacco use among young people and enhance the capacity of countries to design, implement and evaluate tobacco control, and prevention programmes [12, 18]. GYTS is a cross-sectional, nationally representative school-based survey that collects data using standardized methodology for constructing the sample frame, selecting schools and classes and processing data. Following GYTS two-stage sample design, in all of the five countries (the latest available data: Czech Republic (2016), Slovakia (2016), Slovenia (2017), Romania (2017), Lithuania (2018)) schools were selected with a probability proportional to the enrollment size and within these schools classes were randomly chosen. All of the students (aged 11–17 years) from the selected classes were invited to participate in the survey. The overall survey response rates were, as follows: Czech Republic 78.3%, Slovakia 81.7%, Slovenia 68.0%, Romania 88.6%, Lithuania 82.7%. Taking into account the study purpose, so as to assess patterns of susceptibility to tobacco use, the analysis was restricted to never smokers – the participants who responded “no” to the following question “Have you ever tried or experimented with cigarette smoking, even one or two puffs? (44% in Lithuania, 50% in Czech Republic and Slovakia, 68% in Slovenia and Romania). The participants were excluded in case of missing data for a dependent variable (from 0.3% in Czech Republic to 1,8% in Lithuania). That resulted in the following sample size considered in the current analysis: 1997 of 3926 students from Czech Republic, 1998 of 3997 students from Slovakia, 1765 of 2629 students from Slovenia, 3718 of 5409 students from Romania and 1305 of 3030 students from Lithuania.

The Ministry of Health of each country (Public Health Authority of the Slovak Republic) handled scientific, ethical and technical coordination of the study. Following the legal rules in each country, if required, an ethical approval was obtained from the relevant committee and written informed consent was received from all the subjects or, if subjects were under 16, from a parent or legal guardian.

### Questionnaire, dependent and independent variables

GYTS uses a standard, anonymous, self-administered questionnaire that produces estimates on tobacco use (smoking and smokeless), exposure to second hand smoke, tobacco use cessation, access and availability to tobacco, awareness of anti-tobacco information and exposure to tobacco marketing [18, 23]. The questionnaires and databases are publicly available at https://www.cdc.gov/tobacco/global/gtss/gtssdata/index.html [12]. The dependent variable considered in the current study was susceptibility to tobacco product use as adopted from Pierce et al. (1996) [13]. It was based on two questions: “If one of your best friends offered you a tobacco product, would you use it? and “At any time during the next 12 months do you think you will use any form of tobacco?” (in Slovenia the following question was asked: “*At any time during the next 12 months do you think you will smoke tobacco?“*). For each of the questions the following four answers were possible: “Definitely not”, “Probably not”, “Probably yes” and “Definitely yes”. The students who answered “definitely not” to both questions were coded as non-susceptible ones and all the others were considered as susceptible to tobacco use [7].

The independent variables included socio-demographic and economic data: sex, age, parental education (country specific), money available for own expenses during an average week (in local currency); information related to second hand smoking (at home, in public places, parental and peers smoking); knowledge and attitudes regarding tobacco use (harmfulness of active and passive smoking, difficulty of quitting smoking, attractiveness and acceptability of smoking); pro and anti-tobacco media and advertising, and antismoking education provided by the school or family [12]. All the variables were categorical in nature, and most responses were dichotomized using the previously described methods [18].

### Statistical analysis

STATISTICA version 10.0 (Dell Software, Arizona, CA, USA) was used to perform the statistical analysis. Similarly to the previously published studies in this field, the statistical analyses covered several steps [7, 18]. Initially, a descriptive analysis for all the variables involved in the analysis was completed. The univariable and multivariable logistic regression analyses with results being presented as odds ratios (OR) and adjusted odds ratios (AOR) with 95% confidence intervals and *p* values were applied to study the factors linked to susceptibility to tobacco use among youth in the five central and eastern European countries. The analyses were performed separately for each country. In the multivariable analyses, all the variables (*p* < 0.05) were simultaneously included. To test multicollinearity between the variables, the variance inflation factor (VIF) was calculated. All the inflation factors were below 1.5, thus, it can be assumed that collinearity was not present. A *p* value of less than 0.05 was considered statistically significant.

Additionally, the effect estimates were pooled using random-effects meta-analysis following methods described by DerSimonian and Laird [24], which considers both within- and between-study variability. Statistical heterogeneity among included studies was assessed using the Cochran Q test and *I*2 -statistic [25, 26]. All variables were included in the model except parental education and exposure to antismoking media messages (as non significant in any of analyzed countries; *p* > 0.05). *P*-value (Cochnane Q statistics) below 0.05 was considered as significant heterogeneity.

## Results

Characteristics of the students included in the current analysis are summarized in Table S1. Close to one fourth of youth were susceptible to smoking in 4 out of 5 countries (Table 1). A lower percentage of the students classified as susceptible to tobacco use was observed in Romania (16%).

**Table 1:** Susceptibility to tobacco use among never smoking youth from five central and eastern European countries

Characteristics*	Czech Republic *N* = 1997 n (%)	Slovakia *N* = 1998 n (%)	Slovenia *N* = 1765 n (%)	Lithuania *N* = 1305 n (%)	Romania *N* = 3718 n (%)
If one of your best friends offered you a cigarette or another tobacco product, would you use it?					
Definitely not	1639 (82.2)	1630 (81.7)	1529 (86.8)	1091 (84.2)	3309 (89.1)
Probably not	296 (14.8)	283 (14.2)	136 (7.7)	135 (10.4)	284 (7.6)
Probably yes	50 (2.5)	68 (3.4)	80 (4.5)	63 (4.9)	90 (2.4)
Definitely yes	9 (0.5)	13 (0.7)	16 (0.9)	7 (0.5)	31 (0.8)
Missing n (%)**	3 (0.2)	4 (0.2)	4 (0.2)	9 (0.7)	4 (0.1)
At any time during the next 12 months do you think you will use any form of tobacco?^a^					
Definitely not	1667 (83.5)	1713 (85.8)	1467 (83.2)	1101 (84.4)	3302 (88.9)
Probably not	274 (13.7)	228 (11.4)	261 (14.8)	159 (12.2)	291 (7.8)
Probably yes	44 (2.2)	39 (1.9)	29 (1.6)	38 (2.9)	81 (2.2)
Definitely yes	12 (0.6)	17 (0.9)	7 (0.4)	7 (0.5)	40 (1.1)
Missing n (%)**	0 (0.0)	1 (0.1)	1 (0.1%)	0 (0.0)	4 (0.1)
Susceptible to tobacco use^b^					
No	1507 (75.5)	1548 (77.5)	1371 (77.7)	1008 (77.2)	3131 (84.2)
Yes	490 (24.5)	450 (22.5)	394 (22.3)	297 (22.8)	587 (15.8)

^a^in Slovenia the following question was asked: “At any time during the next 12 months do you think you will smoke tobacco?“

^b^chi^2^ test for heterogeneity across countries: *p* < 0.001; country by country comparison: Czech Republic vs Slovakia (*p* = 0.14), Czech Republic vs Slovenia (*p* = 0.11), Czech Republic vs Lithuania (*p* = 0.26), Czech Republic vs Romania (*p* < 0.001), Slovakia vs Slovenia (*p* = 0.88), Slovakia vs Lithuania (*p* = 0.84), Slovakia vs Romania (*p* < 0.001), Slovenia vs Lithuania (*p* = 0.74), Slovenia vs Romania (*p* < 0.001), Lithuania vs Romania (*p* < 0.001)

^*^percentages calculated for observed values

^**^percentages of total number of subjects

### Factors associated with susceptibility to tobacco use among never smokers

Results from the univariable analysis that was run to identify correlates of tobacco use susceptibility are presented in Table S2. In adjusted model, the following factors were identified, consistently across countries, as correlates of tobacco use susceptibility: exposure to passive smoking in public places (AOR from 1.3; *p* = 0.05 in Slovakia to 1.6; *p* < 0.01 in Czech Republic and Romania), peers smoking status (AOR from 1.8 *p* < 0.01 in Slovakia to 2.5; *p* < 0.01 Lithuania), opinion that smoking helped people feel more comfortable at celebrations (AOR from 1.3; *p* = 0.01 in Czech Republic to 1.9; *p* < 0.01 in Lithuania), noticing people using tobacco in mass media (AOR 1.5; *p* < 0.01 in Slovenia and 1.6; *p* < 0.01 in Lithuania), lack of knowledge on harmful effects of passive smoking (AOR 1.8; *p* < 0.01 in Slovakia and 2.4; *p* < 0.01 in Slovenia), lack of antismoking education provided by school (AOR 1.3; *p* < 0.05 in Czech Republic, Slovakia and Slovenia; 1.9; *p* < 0.01 in Lithuania), and family (AOR 1.5; *p* < 0.01 in Slovenia and Romania) (Table 2). Moreover those who believed that smoking makes young people look less attractive (AOR from 0.5; *p* < 0.01 in Romania to 0.7; *p* = 0.05 in Lithuania) and that people who smoke have less friends (AOR 0.7; *p* ≤ 0.06) turned out to be less susceptible to tobacco use initiation.

**Table 2:** Factors associated with susceptibility to tobacco use among never smoking youth from five central and eastern European countries - multivariate logistic regression

Characteristics	Czech Republic *N* = 1947		Slovakia *N* = 1853		Slovenia *N* = 1670		Lithuania *N* = 1236		Romania *N* = 3247	
	AOR (95%CI)	*p*-value	AOR (95%CI)	*p*-value	AOR (95%CI)	*p*-value	AOR (95%CI)	*p*-value	AOR (95%CI)	*p*-value
Sex										
Male (ref.)										
Female	1.38 (1.11–1.72)	< 0.01	NS	NS	1.41 (1.10–1.81)	< 0.01	NS	NS	NS	NS
Age										
13 years or younger (ref.)										
14 years	NS	NS	1.00 (0.77–1.31)	0.98	1.05 (0.75–1.47)	0.79	1.40 (0.96–2.04)	0.09	1.48 (1.18–1.86)	< 0.01
15 years or older	NS	NS	1.29 (0.95–1.73)	0.10	1.10 (0.78–1.53)	0.59	1.25 (0.85–1.85)	0.25	1.12 (0.81–1.54)	0.49
Money available per week for own expenses										
None or little (ref.)										
More	1.46 (1.14–1.86)	< 0.01	1.54 (1.20–1.99)	< 0.01	1.63 (1.26–2.10)	< 0.01	NS	NS	1.51 (1.22–1.86)	< 0.01
Exposure to SHS at home										
No (ref.)										
Yes	1.09 (0.81–1.46)	0.56	1.18 (0.91–1.53)	0.22	NS	NS	NS	NS	1.13 (0.87–1.47)	0.37
Exposure to SHS in public places										
No (ref.)										
Yes	1.57 (1.25–1.98)	< 0.01	1.28 (1.01–1.64)	0.05	1.41 (1.06–1.88)	0.02	1.35 (1.01–1.81)	0.04	1.61 (1.28–2.02)	< 0.01
Parental smoking										
No (none) (ref.)										
Yes (one or both)	1.00 (0.76–1.31)	0.99	NS	NS	N/A	N/A	NS	NS	1.01 (0.79–1.29)	0.94
Peers smoking										
No (ref.)										
Yes	2.24 (1.79–2.79)	< 0.01	1.76 (1.38–2.25)	< 0.01	N/A	N/A	2.47 (1.84–3.33)	< 0.01	2.14 (1.72–2.66)	< 0.01
Seen anyone smoking inside the school or outside on school property										
No (ref.)										
Yes	1.04 (0.82–1.31)	0.76	NS	NS	1.09 (0.83–1.42)	0.54	1.29 (0.96–1.74)	0.09	1.19 (0.96–1.47)	0.11
Knowledge about harmful effects of smoking										
Yes (ref.)										
No	1.53 (0.97–2.42)	0.07	N/A	N/A	N/A	N/A	1.55 (0.92–2.62)	0.10	N/A	N/A
Knowledge about harmful effects of SHS										
Yes (ref.)										
No	1.50 (0.98–2.29)	0.06	1.84 (1.18–2.85)	< 0.01	2.43 (1.50–3.95)	< 0.01	1.39 (0.91–2.12)	0.13	1.14 (0.85–1.53)	0.38
Seen people using tobacco when watched TV, videos or movies										
No (ref.)										
Yes	1.19 (0.92–1.53)	0.19	1.18 (0.93–1.50)	0.18	1.52 (1.11–2.07)	< 0.01	1.59 (1.18–2.16)	< 0.01	1.21 (0.97–1.51)	0.09
Exposure to advertisements or promotions at points of sale										
No (ref.)										
Yes	1.14 (0.91–1.42)	0.25	1.40 (1.09–1.79)	< 0.01	1.49 (1.16–1.92)	< 0.01	NS	NS	0.96 (0.76–1.23)	0.77
School discussion about health effects of smoking										
Yes (ref.)										
No	1.31 (1.05–1.64)	0.02	1.35 (1.06–1.71)	0.01	1.31 (1.02–1.68)	0.03	1.93 (1.43–2.61)	< 0.01	NS	NS
School discussion about the reasons why people use tobacco										
Yes (ref.)										
No	NS	NS	NS	NS	NS	NS	NS	NS	0.77 (0.62–0.95)	0.02
Antismoking education provided by family										
Yes (ref.)										
No	NS	NS	N/A	N/A	1.53 (1.19–1.97)	< 0.01	NS	NS	1.45 (1.15–1.83)	< 0.01
Difficulty of quitting smoking by smoker										
Difficult (ref.)										
Not difficult	NS	NS	NS	NS	NS	NS	NS	NS	1.21 (0.95–1.55)	0.12
Smoking helps people feel more comfortable or less comfortable at celebrations parties or in other social gatherings										
Less comfortable or no differences (ref.)										
More comfortable	1.32 (1.06–1.66)	0.01	1.45 (1.15–1.84)	< 0.01	1.47 (1.15–1.87)	< 0.01	1.88 (1.38–2.56)	< 0.01	1.60 (1.26–2.02)	< 0.01
People who smoke have more or less friends										
No differences (ref.)										
More fiends	N/A	N/A	0.85 (0.61–1.18)	0.34	N/A	N/A	0.78 (0.55–1.10)	0.16	1.36 (1.01–1.82)	0.04
Less friends	N/A	N/A	0.69 (0.49–0.96)	0.03	N/A	N/A	0.66 (0.43–1.02)	0.06	0.74 (0.54–1.01)	0.05
Smoking makes young people look more or less attractive										
No differences (ref.)										
More attractive	N/A	N/A	1.30 (0.84–2.00)	0.23	N/A	N/A	0.91 (0.48–1.75)	0.78	1.12 (0.78–1.61)	0.54
Less attractive	N/A	N/A	0.62 (0.48–0.80)	< 0.01	N/A	N/A	0.72 (0.51–1.01)	0.05	0.54 (0.42–0.70)	< 0.01

*SHS* secondhand smoke, *N/A* data is not available, *NS* not significant in univariable model (at significance level *p* = 0.05), *AOR* adjusted odds ratio

Significant heterogeneity (p for heterogeneity < 0.05) between the countries was observed only for few analyzed variables (Figs. S1, S2, S3, S4 and S5), In Czech Republic and Slovenia significantly higher susceptibility to tobacco use was observed among females as compared to males (AOR 1.4; *p* < 0.01), whereas in Romania opposite pattern, although not significant, was observed (*p* = 0.3). Having more money available for own expenses, positively correlated with smoking suitability in all countries (AOR > 1.5; *p* < 0.01) except Lithuania where youth with more money available tend to be less susceptible to tobacco use (*p* > 0.05). Youth who share the opinion that people who smoke have more friends were more susceptible to smoking in Romania (AOR 1.4; *p* = 0.04) but tend to be less susceptible (although not significantly) in other countries. Exposure to advertisements or promotions at points of sale was significant correlate of tobacco use susceptibility in Slovakia and Slovenia (AOR 1.4 and 1.5 respectively; *p* < 0.05), with moderate heterogeneity between the countries.

The following factors were not significant correlates of tobacco use susceptibility in any of the analyzed countries: parental education, age of the students, parental smoking, exposure to passive smoking at home, presence of the smokers inside school or outside school on school property, knowledge about harmful effects of active smoking, perception of difficulty of quitting by smokers and school discussion of the reasons why people use tobacco (*p* > 0.05).

## Discussion

Our results indicate that nearly a quarter of never cigarette smoking students were susceptible to tobacco use in 4 out of 5 analyzed countries (with slightly lower percentages observed in Romania). Although some differences between the countries were noted, our results present priorities, such as strengthening law, knowledge and attitudes towards smoking, for preventive measures to be taken to decrease percentage of youth who initiate tobacco use. The earlier analysis, based on GYTS in 168 countries, has indicated that around 1 in 8 never-smoking young people worldwide is susceptible to smoking, with the highest percentage of youth declaring smoking susceptibility reported in America and Europe [18]. This is proven by the WHO estimates indicating that smoking prevalence among adolescents and adults in Europe is among the highest in WHO regions [27]. Another assessment based on GYTS data from 25 European countries has also confirmed high susceptibility to smoking among adolescents [28]. Moreover, the study conducted in a rural area of Poland (between 2014 and 2015) based on GYTS questionnaire indicated 22% of never smokers susceptible to tobacco use, which is similar to the results obtained within the current assessments [7]. Data presented in country reports and fact sheets with the consideration of survey weights indicated the highest percentage of never tobacco users susceptible to tobacco use in the future in Czech Republic (22.5%) and the lowest in Romania (14.9%) [12]. Some differences between the studies concerning proportion of those susceptible to smoking can result from several reasons, among which social and cultural norms, tobacco industry influence and legislation as well as preventive activities are the most frequently pointed ones. Moreover, age of a youth, definition of a current non-smoking status (study population restricted to never cigarette smokers vs. never tobacco users), susceptibility to smoking (focusing only on cigarettes or on any tobacco products; types of questions used for assessment) and time period of the study are among other factors. In needs to be underlined that the existing legislation and preventive measures that were in place in the analyzed countries cannot be directly translated into the observed prevalence and, in particular, correlates of tobacco use susceptibility. Many factors can be crucial with this regard such as: youth awareness about existing legislation, its enforcement, time since the law or anti-tobacco national campaign was announced or happened as well as existing social and cultural norms. Moreover, taking into account the overall efforts of the states to combat the tobacco epidemic, the differences in tobacco control measures between the countries are not strongly pronounced. Looking at the countries that have been covered by the current assessment, the strongest tobacco control measures (such as smoke-free policies and anti-tobacco campaign) were observed in Romania and in that country the lowest susceptibility to tobacco initiation was observed as compared to others. To conclude these considerations, all the activities should be multidirectional, multisectorial and sustainable.

In two out of 5 analyzed countries (Czech Republic and Slovenia) females were significantly more susceptible to tobacco use than males, whereas in Romania opposite pattern, although not significant, was observed. Data from other studies are also not fully consistent. In some studies, females have higher risk of tobacco use initiation [15, 29–32], whereas in others, including that conducted in Poland among a rural population, males were more susceptible to smoking [7, 16, 17, 33, 34]. Earlier analyses of GYTS data from European countries (including all 5 countries involved in the current analysis) have also confirmed a similar pattern of smoking susceptibility among never-smoking youth to the one observed in the current assessments [28]. The differences between the countries in the obtained results can be explained by general differences between the countries and the time when the studies were conducted, which in turn, can show target groups for tobacco industry (which are currently females) and how youth are susceptible to tobacco marketing [16].

Pocket money turned out to be a risk factor for tobacco use susceptibility in 4 out of 5 countries covered by our assessment. This finding is in line with GYTS results from Greece, Cyprus or Bangladesh [35–38]. Parents should pay more attention to how their children’s pocket money is used. Furthermore, the role of this factor in susceptibility to smoking needs to be considered together with teenagers’ easy access to tobacco products.

We found that adolescents whose siblings or closest friends are smokers were at higher risk of tobacco use initiation comparing to those with non-smoking peers (this association was observed in all the countries which such data available). In the study conducted in Poland, friends’ smoking status was a stronger predictor of susceptibility to smoking than the parental smoking status [7, 18]. Other studies also indicate that the strongest determinant of current smoking is peer influence [35, 36, 39–43]. Studies indicate that people tend to choose their friends based on shared characteristics, including tobacco use. However, having close friends who use tobacco does not need to mean that they cause a person to use that product. On the other hand, strong commitment not to smoke if offered a cigarette or other tobacco product by a friend is crucial as a protective factor for not starting using tobacco [7]. There is also a need for peer education to help them quit smoking or at least abstinent from smoking in front of non-smokers. Creating a non-smoking fashion among young people seems to be most desirable. This was also proven by our results that indicate that those who declared that tobacco helped people feel more comfortable at celebrations, parties or in other social gatherings and those who emphasized attractiveness of smokers in the peer group (significant for some countries) were at higher risk of tobacco use susceptibility. Moreover, youth who thought that people who smoke had fewer friends and that smoking made youth less attractive had a lower risk of tobacco use susceptibility. So efforts should focus on such perceptions, norms and acceptance of tobacco use.

Exposure to passive smoking in public places was associated with increased susceptibility to tobacco use among the studied populations, which is in agreement with other studies in this field and supports the need to create smoke-free environments worldwide together with effective enforcement of existing legislation [18]. As it was mentioned previously in Romania all public places and in Czech Republic, Slovakia, Lithuania three to five public places were indicated as completely smoke-free [10, 22]; however it needs to be underlined that existing low should go together with its awareness and enforcement.

Lack of knowledge about harmful effects of passive smoking and lack of training in the field of health consequences of tobacco smoking in the school curriculum and lack of antismoking education provided by family was identified as an additional, important factor of susceptibility to tobacco use. Similar results have been observed in other assessments in this field [7, 44]. Imparting knowledge about harmfulness of tobacco has been one of the key tobacco control strategies. According to WHO assessments, some mass media anti-tobacco campaigns were conducted in Slovenia and Romania proceeding the surveys. However, to be fully effective such activities should go together with family and school education and support.

Despite the existing legislation, our results, being in agreement with similar studies in this field, indicate that presence of smoking in movies was associated with an increased risk of tobacco use initiation and tobacco consumption in adolescents [45–47]. Tobacco use by celebrities in the mass media should be eliminated as media play an important role in shaping personality, especially in adolescents and young adults [48]. It has been also shown that susceptibility to tobacco use is influenced by exposure to advertising at points of sale (which in our study was a significant correlate of tobacco susceptibility in Slovakia and Slovenia) [49–51]. Advertising at points of sale still remains a challenge. The results of research suggest that the complete ban on tobacco advertising in points of sale is significantly related to the reduced experimental smoking among adolescents and that this relationship is visible for both sexes [52]. Tobacco industry promotion efforts can be prevented by developing and implementing comprehensive bans along with stronger regulations, including enforcement of law, of tobacco company practices in accordance with WHO Framework Convention on Tobacco Control (FCTC) [53].

The current analysis has several strengths. GYTS is a cross-sectional, nationally representative survey and covers a large number of respondents from an adolescent population, assuring reliability and validity of the results. The protocols and questionnaires in all the analyzed countries were based on GYTS standards developed by experts in the field, which enables a direct comparison between the countries and trends assessments. Moreover, susceptibility to tobacco use was assessed by two questions, which constitute frequently used and reliable measure of predisposition to smoking initiation. Finally, the analysis considers a number of various potential correlates of susceptibility to tobacco including socio-demographic, economic factors, information related to second hand smoking, knowledge and attitudes regarding tobacco use, pro and anti-tobacco media, advertising, and educational issues related to health consequences of smoking.

Limitations of the study also need to be pointed out. Firstly, due to the cross-sectional nature of the study, claims of causation cannot be made about the observed relationships between susceptibility to tobacco use and the studied variables. Secondly, all the estimates in our assessment were based on self-reports, which might be affected by reporting bias. Moreover, some differences in the response rates exists between the countries (from 68% in Slovenia to 89% in Romania), which may bias the obtained results. The willingness to participate in the study can be determined by each country’s socio-cultural norms, the level of trust and acceptance of being interviewed on sensitive issues. Moreover, some missing data for independent variables occurred (and they were not imputed in the analysis). We are aware of the fact that a high amount of missing data can affect the results and conclusions from the study. However, we want to point out that for majority of the individual variables, the missing data were below 1% and all, except for parental education, were below 4%. Parental education variables were not included in multivariable model, thus, do not impact the final sample size. GYTS questionnaire contains some core questions. Additional questions could be selected or added by the experts depending on the country or specific issues studied. That is why some of the variables were not available in all the analyzed populations. It also needs to be underlined that some questions, although similar in their meaning, were not asked exactly the same in all the countries, which can also impact the obtained results. The years in which the surveys were conducted in each of the analyzed countries, were as close as possible. Nevertheless, it cannot be excluded that the fact of different years of data collection may have influence on the associations noted in the study. Additionally, considering other papers in this field we decided to restrict our study population to non-cigarette smokers (but some part of such students could use other tobacco products). This creates the opportunity for other researchers to look more closely at the population of non-tobacco users. Finally, our analysis did not control for other substances use such as alcohol or illicit drug, which are also indicated to be associated with smoking. Despite the limitations stated above, this study provides an important insight into the prevalence and factors associated with susceptibility to tobacco use in central and eastern European countries. These correlates are crucial for effectiveness of prevention strategies to be taken among youth.

## Conclusions

A high proportion of youth from central and eastern European countries is susceptible to tobacco use. Social factors, and those related to educational and policy issues as well as to attitudes regarding tobacco use were strongly, and consistently across countries, correlated with tobacco use susceptibility. Slight differences in susceptibility to tobacco use between the countries were related to: sex, money available for own expenses, exposure to advertisements or promotions at points of sale and opinion that people who smoke have more friends. These factors should be considered when designing and implementing anti-tobacco activities among young people.

## Supplementary Information

**Additional file 1: Table S1.** Susceptibility to tobacco use among never smokers from five central and eastern European countries. **Table S2.** Factors associated with susceptibility to tobacco use among never smoking youth from five central and eastern European countries - univariable logistic regression. **Fig. S1.** Forest plots showing adjusted associations of selected sociodemographic factors and susceptibility to tobacco use among never smoking youth from five central and eastern European countries. Country-specific odds ratio (OR) and 95% confidence interval (CI) were estimated by multivariable logistic regression models, and overall OR and 95% CI were estimated by random-effects meta-analysis by country. The following factors were included in the model: sex, age, money available for own expenses, SHS exposure at home and in public places, parental and peers smoking, knowledge about harmfulness of active and passive smoking, difficulty of quitting smoking, attractiveness and popularity of smoking, antismoking education provided by the school or family, tobacco advertising at point of sale, noticing people smoking at school and in movies. I^2^, percentage of the total variability attributable to between-country heterogeneity; p, *p* value of heterogeneity using the Cochran’s Q test. **Fig. S2.** Forest plots showing adjusted associations of selected factors related to SHS exposure and susceptibility to tobacco use among never smoking youth from five central and eastern European countries. Country-specific odds ratio (OR) and 95% confidence interval (CI) were estimated by multivariable logistic regression models, and overall OR and 95% CI were estimated by random-effects meta-analysis by country. The following factors were included in the model: sex, age, money available for own expenses, SHS exposure at home and in public places, parental and peers smoking, knowledge about harmfulness of active and passive smoking, difficulty of quitting smoking, attractiveness and popularity of smoking, antismoking education provided by the school or family, pro-tobacco media and advertising. I^2^, percentage of the total variability attributable to between-country heterogeneity; p, *p* value of heterogeneity using the Cochran’s Q test. **Fig. S3.** Forest plots showing adjusted associations of noticing tobacco advertising at point of sale, people smoking at school and in movies and susceptibility to tobacco use among never smoking youth from five central and eastern European countries. Country-specific odds ratio (OR) and 95% confidence interval (CI) were estimated by multivariable logistic regression models, and overall OR and 95% CI were estimated by random-effects meta-analysis by country. The following factors were included in the model: sex, age, money available for own expenses, SHS exposure at home and in public places, parental and peers smoking, knowledge about harmfulness of active and passive smoking, difficulty of quitting smoking, attractiveness and popularity of smoking, antismoking education provided by the school or family, pro-tobacco media and advertising. I^2^, percentage of the total variability attributable to between-country heterogeneity; p, *p* value of heterogeneity using the Cochran’s Q test. **Fig. S4.** Forest plots showing adjusted associations of knowledge about harmfulness of active and passive smoking, difficulty of quitting smoking, and susceptibility to tobacco use among never smoking youth from five central and eastern European countries. Country-specific odds ratio (OR) and 95% confidence interval (CI) were estimated by multivariable logistic regression models, and overall OR and 95% CI were estimated by random-effects meta-analysis by country. The following factors were included in the model: sex, age, money available for own expenses, SHS exposure at home and in public places, parental and peers smoking, knowledge about harmfulness of active and passive smoking, difficulty of quitting smoking, attractiveness and popularity of smoking, antismoking education provided by the school or family, tobacco advertising at point of sale, noticing people smoking at school and in movies. I^2^, percentage of the total variability attributable to between-country heterogeneity; p, *p* value of heterogeneity using the Cochran’s Q test. **Fig. S5.** Forest plots showing adjusted associations of attractiveness and popularity of smoking and susceptibility to tobacco use among never smoking youth from five central and eastern European countries. Country-specific odds ratio (OR) and 95% confidence interval (CI) were estimated by multivariable logistic regression models, and overall OR and 95% CI were estimated by random-effects meta-analysis by country. The following factors were included in the model: sex, age, money available for own expenses, SHS exposure at home and in public places, parental and peers smoking, knowledge about harmfulness of active and passive smoking, difficulty of quitting smoking, attractiveness and popularity of smoking, antismoking education provided by the school or family, tobacco advertising at point of sale, noticing people smoking at school and in movies. I^2^, percentage of the total variability attributable to between-country heterogeneity; p, *p* value of heterogeneity using the Cochran’s Q test.

## Data Availability

The questionnaires and databases are publicly available at: Global Tobacco Surveillance System Data (GTSSData): https://www.cdc.gov/tobacco/global/gtss/gtssdata/index.htmlAccessed 10 October 2020.
