# Supplementary material for: Susceptibility to tobacco use and associated factors among youth in five central and eastern European countries
Source: BMC Public Health. 2022 Jan 11;22:72. doi: 10.1186/s12889-022-12493-6 (PMC8753878; doi:10.1186/s12889-022-12493-6)
Supplement: Supplementary file 1 — Additional file 1: Table S1. Susceptibility to tobacco use among never smokers from five central and eastern European countries. Table S2. Factors associated with susceptibility to tobacco use among never smoking youth from five central and eastern European countries - univariable logistic regression. Fig. S1. Forest plots showing adjusted associations of selected sociodemographic factors and susceptibility to tobacco use among never smoking youth from five central and eastern European countries. Country-specific odds ratio (OR) and 95% confidence interval (CI) were estimated by multivariable logistic regression models, and overall OR and 95% CI were estimated by random-effects meta-analysis by country. The following factors were included in the model: sex, age, money available for own expenses, SHS exposure at home and in public places, parental and peers smoking, knowledge about harmfulness of active and passive smoking, difficulty of quitting smoking, attractiveness and popularity of smoking, antismoking education provided by the school or family, tobacco advertising at point of sale, noticing people smoking at school and in movies. I2, percentage of the total variability attributable to between-country heterogeneity; p, p value of heterogeneity using the Cochran’s Q test. Fig. S2. Forest plots showing adjusted associations of selected factors related to SHS exposure and susceptibility to tobacco use among never smoking youth from five central and eastern European countries. Country-specific odds ratio (OR) and 95% confidence interval (CI) were estimated by multivariable logistic regression models, and overall OR and 95% CI were estimated by random-effects meta-analysis by country. The following factors were included in the model: sex, age, money available for own expenses, SHS exposure at home and in public places, parental and peers smoking, knowledge about harmfulness of active and passive smoking, difficulty of quitting smoking, attractiveness and popularity [file 12889_2022_12493_MOESM1_ESM.pdf]

Susceptibility to tobacco use and associated factors among youth in five central and eastern European countries

Kinga Polanska*, Malgorzata Znyk, Dorota Kaleta

Department of Hygiene and Epidemiology, Medical University of Lodz, Poland

*Corresponding author:

Kinga Polanska, MPH, Ph.D.

Department of Hygiene and Epidemiology,

Medical University of Lodz,

7/9 Zeligowskiego Str., 90-647 Lodz, Poland

kinga.polanska@umed.lodz.pl; Tel.: +48-426-781-688

Email addresses:

Kinga Polanska: kinga.polanska@umed.lodz.pl

Malgorzata Znyk: malgorzata.znyk@umed.lodz.pl

Dorota Kaleta: dorota.kaleta@umed.lodz.pl

Table S1. Susceptibility to tobacco use among never smokers from five central and eastern European countries

Characteristics[*]	Czech Republic		Slovakia		Slovenia		Lithuania		Romania	
	N=1997 (100%)	n=490 (24.5%)	N=1998 (100%)	n=450 (22.5%)	N=1765 (100%)	n=394 (22.3%)	N=1305 (100%)	n=297 (22.8%)	N=3718 (100%)	n=587 (15.8%)
Sex										
Male	953 (47.7)	210 (22.0)	946 (47.5)	208 (22.0)	812 (46.2)	154 (19.0)	581 (44.5)	126 (21.7)	1723 (46.5)	283 (16.4)
Female	1044 (52.3)	280 (26.8)	1046 (52.5)	241 (23.0)	946 (53.8)	236 (25.0)	724 (55.5)	171 (23.6)	1986 (53.5)	303 (15.3)
Missing[**]	0 (0.0)		6 (0.3)		7 (0.4)		0 (0.0)		9 (0.2)	
Age										
13 years or younger	922 (46.2)	210 (22.8)	931 (46.6)	182 (19.5)	408 (23.2)	77 (18.9)	347 (26.6)	63 (18.2)	2112 (56.9)	266 (12.6)
14 years	604 (30.2)	154 (25.5)	642 (32.1)	150 (23.4)	595 (33.8)	119 (20.0)	481 (36.9)	121 (25.2)	1130 (30.4)	229 (20.3)
15 years or older	471 (23.6)	126 (26.8)	424 (21.2)	118 (27.8)	756 (43.0)	196 (25.9)	476 (36.5)	112 (23.5)	472 (12.7)	92 (19.5)
Missing[**]	0 (0.0)		1 (0.1)		6 (0.3)		1 (0.1)		4 (0.1)	
Money available per week for own expenses										
None or little	1522 (76.5)	341 (22.4)	764 (38.8)	130 (17.0)	855 (48.8)	139 (16.3)	335 (25.8)	79 (24.7)	2080 (56.1)	254 (12.2)
More	467 (23.5)	145 (31.0)	1205 (61.2)	315 (26.1)	896 (51.2)	252 (28.1)	965 (74.2)	216 (22.4)	1625 (43.9)	333 (20.5)
Missing[**]	8 (0.4)		29 (1.5)		14 (0.8)		5 (0.4)		13 (0.3)	
Mother's education										
Below College or University	N/A	N/A	958 (59.8)	220 (19.2)	N/A	N/A	213 (22.5)	57 (31.5)	1592 (54.0)	248 (15.6)
College or University			645 (40.2)	163 (25.3)			734 (77.5)	168 (22.4)	1355 (46.0)	228 (16.8)
Missing or I don't know[**]			395 (19.8)				358 (27.4)		771 (20.7)	
Father's education										
Below College or University	N/A	N/A	999 (65.8)	230 (23.0)	N/A	N/A	224 (25.8)	53 (23.7)	1436 (51.7)	225 (16.5)
College or University			520 (34.2)	128 (24.6)			643 (74.2)	155 (24.1)	1343 (48.3)	227 (22.9)
Missing or I don't know[**]			479 (24.0)				438 (24.8)		939 (25.3)	
Exposure to SHS at home										
No	1552 (77.8)	358 (23.1)	1467 (74.1)	300 (20.5)	1341 (76.2)	287 (21.4)	894 (68.6)	192 (21.5)	2698 (72.9)	385 (14.3)
Yes	444 (22.2)	131 (29.5)	514 (25.9)	142 (27.6)	419 (23.8)	107 (25.5)	409 (31.4)	105 (25.7)	1003 (27.1)	198 (19.7)
Missing[**]	1 (0.1)		17 (0.9)		5 (0.3)		2 (0.2)		17 (0.5)	
Exposure to SHS in public places										
No	1361 (68.2)	279 (20.5)	1166 (58.8)	215 (18.4)	1413 (80.4)	287 (20.3)	704 (54.1)	138 (19.6)	2750 (74.2)	355 (12.9)
Yes	634 (31.8)	210 (33.1)	818 (41.2)	230 (28.1)	345 (19.6)	103 (29.9)	597 (45.9)	157 (26.3)	954 (25.8)	224 (23.5)
Missing[**]	2 (0.1)		14 (0.7)		7 (0.4)		4 (0.3)		14 (0.4)	
Parental smoking										
No (none)	1287 (65.0)	290 (22.5)	1263 (63.5)	274 (21.7)	N/A	N/A	773 (61.3)	167 (21.6)	1940 (53.4)	269 (13.9)
Yes (one or both)	693 (35.0)	193 (27.9)	725 (36.5)	171 (23.6)			489 (38.7)	120 (24.5)	1696 (46.6)	302 (17.8)
Missing or don't know[**]	17 (2.3)		10 (0.5)				43 (3.3)		82 (2.2)	
Peers smoking										
No	1298 (65.1)	238 (18.3)	1051 (53.0)	169 (16.1)	N/A	N/A	699 (53.6)	104 (14.9)	2306 (62.4)	239 (10.4)
Yes	697 (34.9)	251 (36.0)	933 (47.0)	277 (29.7)			605 (46.4)	192 (31.7)	1387 (37.6)	341 (24.6)
Missing[**]	2 (0.1)		14 (0.7)				1 (0.1)		25 (0.7)	
Seen anyone smoking inside the school or outside on school property										
Yes	611 (30.7)	168 (27.5)	660 (33.1)	161 (24.4)	829 (47.2)	212 (25.6)	651 (50.1)	170 (26.1)	1548 (42.5)	274 (17.7)
No	1382 (69.3)	320 (23.2)	1333 (66.9)	289 (21.7)	928 (52.8)	182 (19.6)	649 (49.9)	124 (19.1)	2092 (57.5)	287 (13.7)
Missing[**]	4 (0.2)		5 (0.3)		8 (0.5)		5 (0.4)		78 (2.1)	
Knowledge about harmful effects of smoking										
No	108 (5.4)	36 (33.3)	N/A	N/A	N/A	N/A	102 (7.9)	35 (34.3)	N/A	N/A
Yes	1886 (94.6)	453 (24.0)					1191 (92.1)	257 (21.6)		
Missing[**]	3 (0.2)						12 (0.9)			
Knowledge about harmful effects of SHS										

No	129 (6.5)	44 (34.1)	124 (6.2)	45 (36.3)	94 (5.3)	35 (37.2)	160 (12.3)	49 (30.6)	572 (15.5)	136 (23.8)
Yes	1866 (93.5)	446 (23.9)	1868 (93.8)	404 (21.6)	1667 (94.7)	358 (21.5)	1144 (87.7)	248 (21.7)	3120 (84.5)	445 (14.3)
Missing**	2 (0.1)		6 (0.3)		4 (0.2)		1 (0.1)		26 (0.7)	
Seen people using tobacco when watched TV, videos or movies										
Yes	1423 (71.3)	371 (26.1)	1018 (51.5)	250 (24.6)	1323 (75.0)	323 (24.4)	724 (56.2)	185 (25.6)	1986 (54.0)	335 (16.9)
No	572 (28.7)	118 (20.6)	960 (48.5)	197 (20.5)	440 (25.0)	71 (16..1)	565 (43.8)	109 (19.3)	1694 (46.0)	239 (14.1)
Missing**	2 (0.1)		20 (1.0)		2 (0.1)		16 (1.2)		38 (1.0)	
Exposure to advertisements or promotions at points of sale										
Yes	878 (44.0)	240 (27.3)	603 (30.4)	169 (28.0)	543 (30.8)	156 (28.7)	226 (17.4)	58 (25.7)	923 (25.0)	171 (18.5)
No	1117 (56.0)	248 (22.2)	1383 69.6)	281 (20.3)	1218 (69.2)	237 (19.5)	1076 (82.6)	239 (22.2)	2763 (75.0)	403 (14.6)
Missing**	2 (0.1)		12 (0.6)		4 (0.2)		3 (0.2)		32 (0.9)	
Exposure to antismoking media messages										
Yes	949 (47.7)	229 (24.1)	932 (47.3)	191 (20.5)	787 (44.8)	184 (23.4)	602 (46.7)	127 (21.1)	2213 (60.4)	338 (15.3)
No	1042 (52.3)	259 (24.9)	1040 (52.7)	250 (24.0)	969 (55.2)	206 (21.3)	687 (53.5)	162 (23.6)	1452 (39.6)	227 (15.6)
Missing**	6 (0.3)		26 (1.3)		9 (0.5)		16 (1.2)		53 (1.4)	
School discussion about health effects of smoking										
Yes	872 (43.8)	189 (21.7)	879 (44.1)	177 (20.1)	741 (42.1)	140 (18.9)	823 (63.1)	149 (18.1)	1550 (42.0)	239 (15.4)
No	1119 (56.2)	297 (26.5)	1112 (55.9)	272 (24.5)	1018 (57.9)	253 (24.9)	481 (36.9)	147 (30.6)	2137 (58.0)	327 (15.3)
Missing**	6 (0.3)		7 (0.4)		6 (0.3)		1 (0.1)		31 (0.8)	
School discussion about the reasons why people use tobacco										
Yes	592 (29.7)	142 (24.0)	648 (32.6)	144 (22.2)	443 (25.3)	103 (23.3)	506 (38.8)	108 (21.3)	1361 (36.9)	243 (17.9)
No	1398 (70.3)	346 (24.8)	1340 (67.4)	304 (22.7)	1310 (74.7)	289 (22.1)	798 (61.2)	189 (23.7)	2328 (63.1)	331 (14.2)
Missing**	7 (0.4)		10 (0.5)		12 (0.7)		1 (0.1)		29 (0.8)	
Antismoking education provided by family										
Yes	1268 (63.8)	298 (23.5)	N/A	N/A	1210 (69.5)	238 (19.7)	765 (59.7)	167 (21.8)	2532 (69.4)	353 (13.9)
No	719 (36.2)	190 (26.4)			530 (30.5)	150 (28.3)	517 (40.3)	123 (23.8)	1116 (30.6)	195 (17.5)
Missing**	10 (0.5)				25 (1.4)		23 (1.8)		70 (1.9)	
Difficulty of quitting smoking by smoker										
Not difficult	153 (7.7)	43 (28.1)	328 (16.5)	80 (24.4)	281 (16.1)	67 (23.8)	502 (38.5)	117 (23.3)	922 (24.9)	188 (20.4)
Difficult	1843 (92.3)	447 (24.3)	1658 (83.5)	364 (21.9)	1468 (83.9)	323 (22.0)	801 (61.5)	179 (22.3)	2783 (75.1)	394 (14.2)
Missing**	1 (0.1)		12 (0.6)		16 (0.9)		2 (0.2)		13 (0.3)	
Smoking helps people feel more comfortable or less comfortable at celebrations parties or in other social gatherings										
More comfortable	723 (36.4)	202 (27.9)	862 (44.0)	220 (25.5)	849 (48.9)	220 (25.9)	348 (27.0)	110 (31.6)	792 (21.8)	179 (22.6)
Less comfortable or no differences	1264 (63.6)	286 (22.6)	1096 (56.0)	220 (20.1)	886 (51.1)	165 (18.6)	941 (73.0)	179 (19.0)	2840 (78.2)	376 (13.2)
Missing**	10 (0.5)		40 (2.0)		30 (1.7)		16 (1.2)		86 (2.3)	
People who smoke have more or less friends										
More fiends	N/A	N/A	330 (16.7)	75 (22.7)	N/A	N/A	425 (32.7)	95 (22.4)	576 (15.8)	122 (21.2)
Less friends			432 (21.9)	68 (15.7)			267 (20.6)	42 (15.7)	1067 (29.3)	104 (9.8)
No differences			1211 (61.4)	297 (24.5)			606 (46.&)	157 (25.9)	2002 (54.9)	338 (16.9)
Missing**			25 (1.3)				7 (0.5)		73 (2.0)	
Smoking makes young people look more or less attractive										
More attractive	N/A	N/A	63 (3.4)	54 (33.1)	N/A	N/A	75 (5.8)	20 (26.7)	317 (8.7)	82 (25.9)
Less attractive			1228 (65.9)	225 (18.3)			874 (67.6)	170 (19.5)	1727 (47.2)	169 (9.8)
No differences			572 (30.7)	154 (26.9)			344 (26.6)	105 (30.5)	1618 (44.2)	311 (19.2)
Missing**			35 (1.8)				12 (0.9)		56 (1.5)	

*percentages calculated for observed values; **percentages of total number of subjects

SHS – secondhand smoke

N/A – data is not available

Table S2. Factors associated with susceptibility to tobacco use among never smoking youth from five central and eastern European countries - univariable logistic regression

Characteristics	Czech Republic N=1997		Slovakia N=1998		Slovenia N=1765		Lithuania N=1305		Romania N=3718	
	OR (95 CI)	p-value	OR (95 CI)	p-value	OR (95 CI)	p-value	OR (95 CI)	p-value	OR (95 CI)	p-value
Sex										
Male (ref.)										
Female	1.30 (1.06-1.59)	0.01	1.06 (0.86-1.31)	0.57	1.42 (1.13-1.79)	<0.01	1.12 (0.86-1.45)	0.41	0.92 (0.77-1.09)	0.33
Age										
13 years or younger (ref.)										
14 years	1.16 (0.91-1.47)	0.22	1.25 (0.98-1.60)	0.07	1.07 (0.78-1.48)	0.66	1.52 (1.08-2.13)	0.02	1.76 (1.45-2.14)	<0.01
15 years or older	1.24 (0.96-1.60)	0.10	1.59 (1.21-2.07)	<0.01	1.50 (1.12-2.02)	<0.01	1.39 (0.98-1.96)	0.06	1.68 (1.29-2.18)	<0.01
Money available per week for own expenses										
None or little (ref.)										
More	1.56 (1.24-1.96)	<0.01	1.73 (1.37-2.17)	<0.01	2.02 (1.60-2.54)	<0.01	0.93 (0.70-1.25)	0.65	1.85 (1.55-2.21)	0.93
Mother's education										
Below College or University (ref.)										
College or University	N/A		1.13 (0.90-1.43)	0.29	N/A		0.81 (0.57-1.15)	0.24	1.10 (0.90-1.33)	0.36
Father's education										
Below College or University (ref.)										
College or University	N/A		1.09 (0.85-1.40)	0.49	N/A		1.02 (0.72-1.47)	0.89	1.09 (0.89-1.34)	0.38
Exposure to SHS at home										
No (ref.)										
Yes	1.40 (1.10-1.77)	0.01	1.48 (1.18-1.87)	<0.01	1.26 (0.98-1.63)	0.08	1.26 (0.96-1.66)	0.09	1.48 (1.22-1.79)	<0.01
Exposure to SHS in public places										
No (ref.)										
Yes	1.92 (1.55-2.37)	<0.01	1.73 (1.40-2.14)	<0.01	1.67 (1.28-2.18)	<0.01	1.46 (1.13-1.90)	<0.01	2.07 (1.72-2.49)	<0.01
Parental smoking										
No (none) (ref.)										
Yes (one or both)	1.33 (1.07-1.64)	0.01	1.11 (0.90-1.38)	0.33	N/A		1.18 (0.90-1.54)	0.23	1.35 (1.13-1.61)	<0.01
Peers smoking										
No (ref.)										
Yes	2.51 (2.03-3.09)	<0.01	2.20 (1.77-2.74)	<0.01	N/A		2.66 (2.03-3.48)	<0.01	2.82 (2.35-3.38)	<0.01
Seen anyone smoking inside the school or outside on school property										
No (ref.)										
Yes	1.26 (1.01-1.56)	0.04	1.17 (0.93-1.45)	0.17	1.41 (1.12-1.76)	<0.01	1.50 (1.15-1.94)	<0.01	1.35 (1.13-1.62)	<0.01
Knowledge about harmful effects of smoking										
Yes (ref.)										
No	1.58 (1.05-2.39)	0.03	N/A		N/A		1.90 (1.23-2.92)	<0.01	N/A	
Knowledge about harmful effects of SHS										
Yes (ref.)										
No	1.65 (1.13-2.41)	0.01	2.06 (1.41-3.03)	<0.01	2.17 (1.40-3.35)	<0.01	1.59 (1.11-2.30)	0.01	1.88 (1.51-2.33)	<0.01
Seen people using tobacco when watched TV, videos or movies										
No (ref.)										
Yes	1.36 (1.07-1.72)	0.01	1.26 (1.02-1.56)	0.03	1.67 (1.26-2.23)	<0.01	1.44 (1.10-1.88)	0.01	1.24 (1.03-1.48)	0.03
Exposure to advertisements or promotions at points of sale										

No (ref.)										
Yes	1.32 (1.07-1.62)	0.01	1.53 (1.22-1.90)	<0.01	1.67 (1.32-2.11)	<0.01	1.21 (0.87-1.68)	0.26	1.33 (1.09-1.62)	<0.01
Exposure to antismoking media messages										
Yes (ref.)										
No	1.04 (0.85-1.28)	0.71	1.23 (0.99-1.52)	0.06	0.88 (0.71-1.11)	0.29	1.15 (0.89-1.50)	0.29	1.03 (0.86-1.23)	0.77
School discussion about health effects of smoking										
Yes (ref.)										
No	1.31 (1.06-1.61)	0.01	1.28 (1.04-1.59)	0.02	1.42 (1.13-1.79)	<0.01	1.99 (1.53-2.59)	<0.01	0.99 (0.83-1.19)	0.92
School discussion about the reasons why people use tobacco										
Yes (ref.)										
No	1.04 (0.83-1.30)	0.72	1.03 (0.82-1.29)	0.82	0.93 (0.72-1.21)	0.60	1.14 (0.87-1.50)	0.33	0.76 (0.64-0.91)	<0.01
Antismoking education provided by family										
Yes (ref.)										
No	1.17 (0.95-1.44)	0.15	N/A		1.61 (1.27-2.04)	<0.01	1.12 (0.86-1.46)	0.41	1.31 (1.08-1.58)	<0.01
Difficulty of quitting smoking by smoker										
Difficult (ref.)										
Not difficult	1.22 (0.84-1.76)	0.29	1.15 (0.87-1.51)	0.33	1.11 (0.82-1.50)	0.50	1.06 (0.81-1.38)	0.69	1.55 (1.28-1.88)	<0.01
Smoking helps people feel more or less comfortable at celebrations parties or in other social gatherings										
Less comfortable or no differences (ref.)										
More comfortable	1.33 (1.08-1.63)	0.01	1.36 (1.10-1.69)	<0.01	1.53 (1.22-1.92)	<0.01	1.97 (1.49-2.60)	<0.01	1.91 (1.57-2.33)	<0.01
People who smoke have more or less friends										
No differences (ref.)										
More fiends	N/A		0.91 (0.68-1.21)	0.50	N/A		0.82 (0.61-1.10)	0.19	1.32 (1.05-1.67)	0.02
Less friends			0.57 (0.43-0.77)	<0.01			0.53 (0.37-0.78)	<0.01	0.53 (0.42-0.67)	<0.01
Smoking makes young people look more or less attractive										
No differences (ref.)										
More attractive	N/A		1.34 (0.92-1.96)	0.12	N/A		0.83 (0.47-1.45)	0.51	1.47 (1.11-1.94)	<0.01
Less attractive			0.61 (0.48-0.77)	<0.01			0.55 (0.41-0.73)	<0.01	0.46 (0.37-0.56)	<0.01

SHS – secondhand smoke
N/A – data is not available

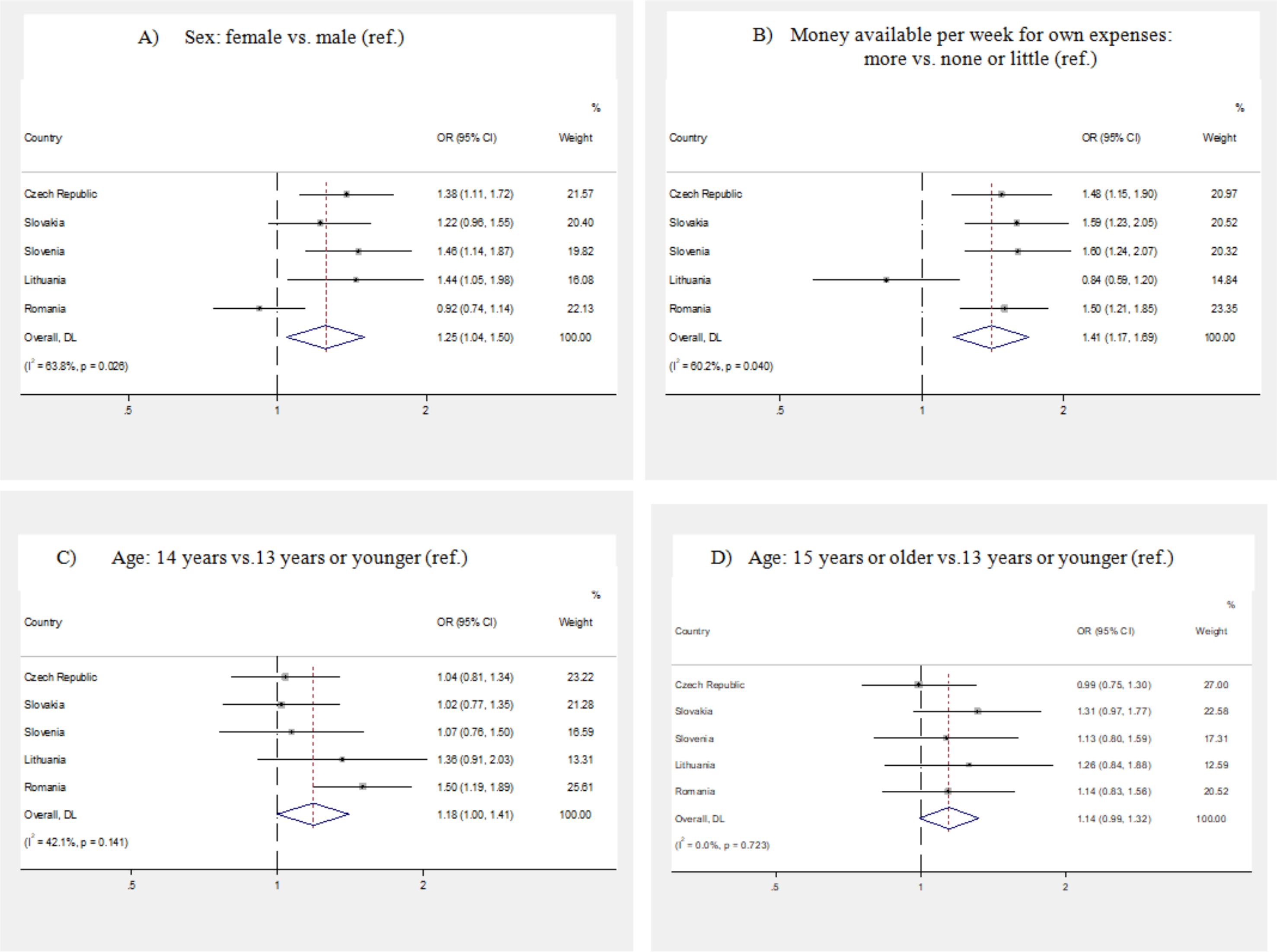

Fig. S1. Forest plots showing adjusted associations of selected sociodemographic factors and susceptibility to tobacco use among never smoking youth from five central and eastern European countries. Country-specific odds ratio (OR) and 95% confidence interval (CI) were estimated by multivariable logistic regression models, and overall OR and 95% CI were estimated by random-effects meta-analysis by country. The following factors were included in the model: sex, age, money available for own expenses, SHS exposure at home and in public places, parental and peers smoking, knowledge about harmfulness of active and passive smoking, difficulty of quitting smoking, attractiveness and popularity of smoking, antismoking education provided by the school or family, tobacco advertising at point of sale, noticing people smoking at school and in movies. I^2, percentage of the total variability attributable to between-country heterogeneity; p, p value of heterogeneity using the Cochran's Q test.

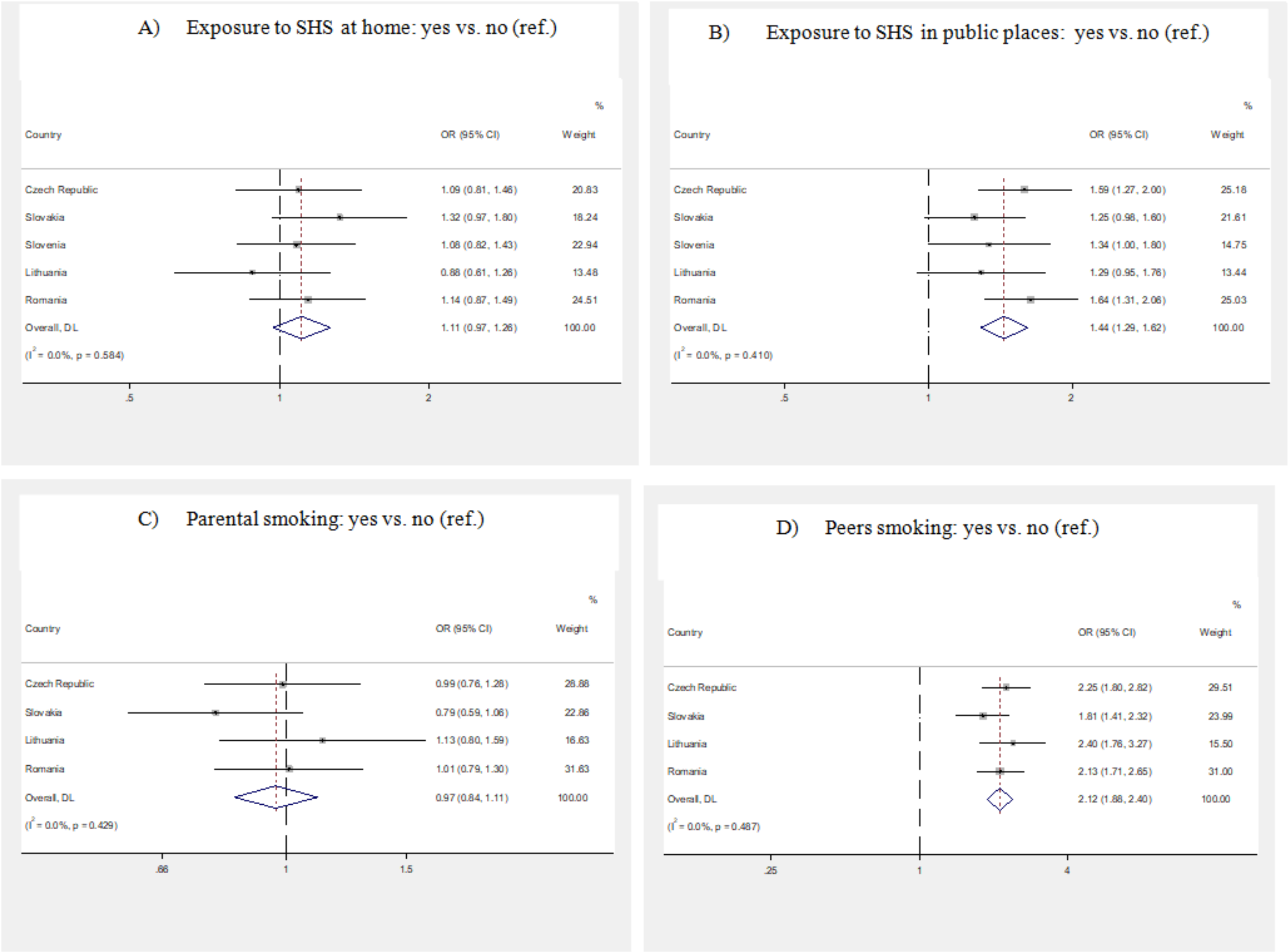

Fig. S2. Forest plots showing adjusted associations of selected factors related to SHS exposure and susceptibility to tobacco use among never smoking youth from five central and eastern European countries. Country-specific odds ratio (OR) and 95% confidence interval (CI) were estimated by multivariable logistic regression models, and overall OR and 95% CI were estimated by random-effects meta-analysis by country. The following factors were included in the model: sex, age, money available for own expenses, SHS exposure at home and in public places, parental and peers smoking, knowledge about harmfulness of active and passive smoking, difficulty of quitting smoking, attractiveness and popularity of smoking, antismoking education provided by the school or family, pro-tobacco media and advertising. I^2, percentage of the total variability attributable to between-country heterogeneity; p, p value of heterogeneity using the Cochran's Q test.

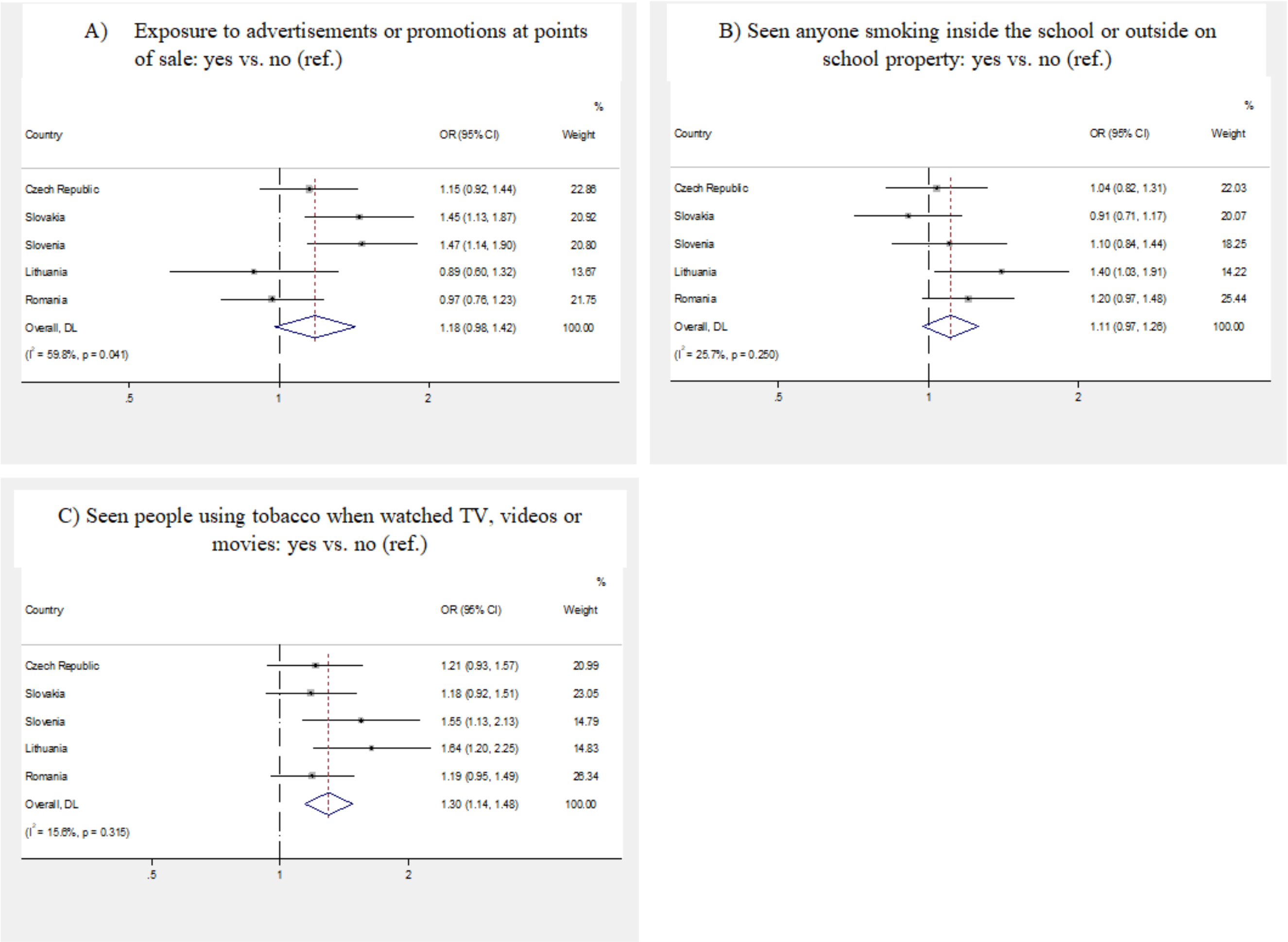

Fig. S3. Forest plots showing adjusted associations of noticing tobacco advertising at point of sale, people smoking at school and in movies and susceptibility to tobacco use among never smoking youth from five central and eastern European countries. Country-specific odds ratio (OR) and 95% confidence interval (CI) were estimated by multivariable logistic regression models, and overall OR and 95% CI were estimated by random-effects meta-analysis by country. The following factors were included in the model: sex, age, money available for own expenses, SHS exposure at home and in public places, parental and peers smoking, knowledge about harmfulness of active and passive smoking, difficulty of quitting smoking, attractiveness and popularity of smoking, antismoking education provided by the school or family, pro-tobacco media and advertising. I^2, percentage of the total variability attributable to between-country heterogeneity; p, p value of heterogeneity using the Cochran's Q test.

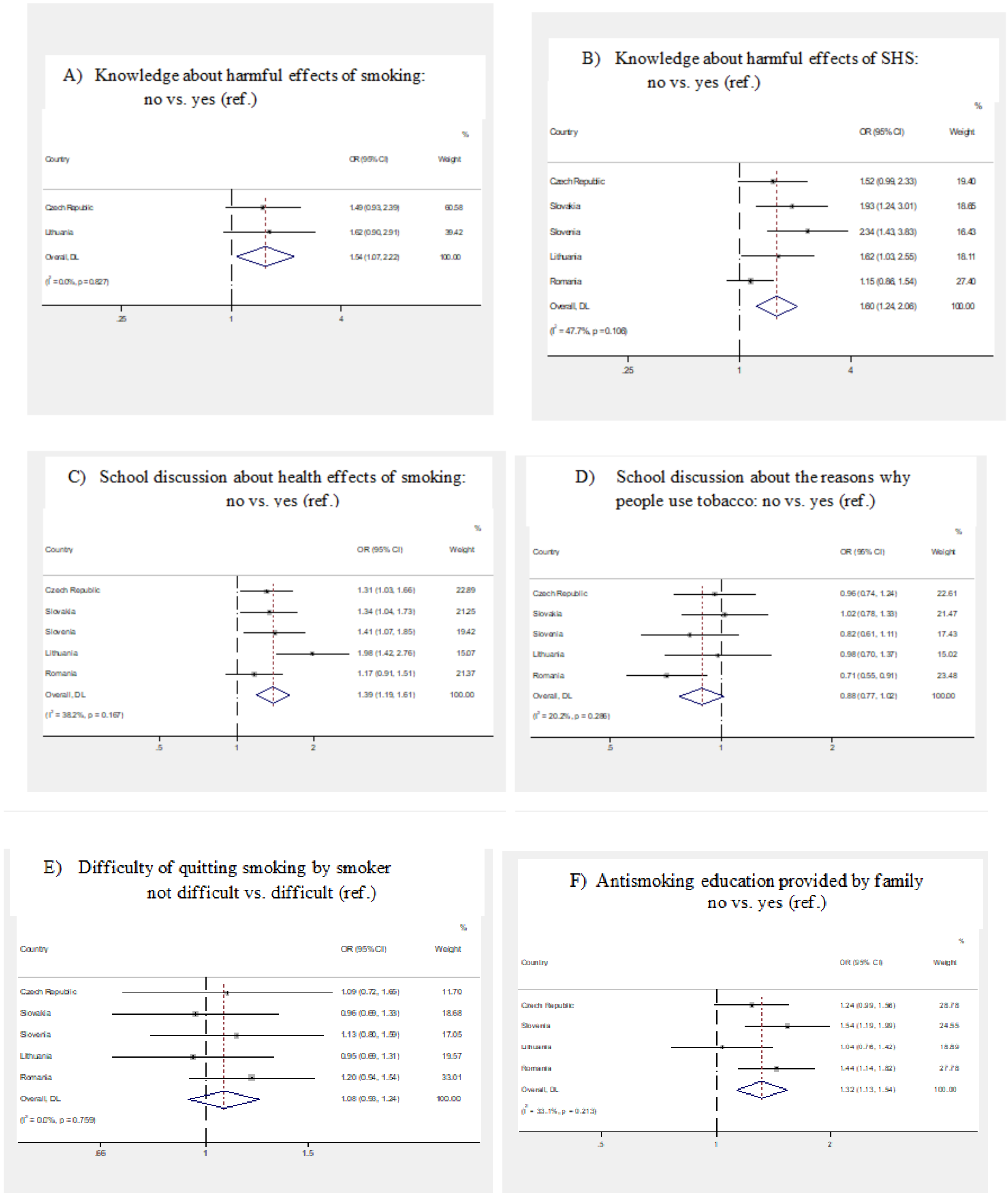

Fig. S4. Forest plots showing adjusted associations of knowledge about harmfulness of active and passive smoking, difficulty of quitting smoking, and susceptibility to tobacco use among never smoking youth from five central and eastern European countries. Country-specific odds ratio (OR) and 95% confidence interval (CI) were estimated by multivariable logistic regression models, and overall OR and 95% CI were estimated by random-effects meta-analysis by country. The following factors were included in the model: sex, age, money available for own expenses, SHS exposure at home and in public places, parental and peers smoking, knowledge about harmfulness of active and passive smoking, difficulty of quitting smoking, attractiveness and popularity of smoking, antismoking education provided by the school or family, tobacco advertising at point of sale, noticing people smoking at school and in movies. I^2, percentage of the total variability attributable to between-country heterogeneity; p, p value of heterogeneity using the Cochran's Q test.

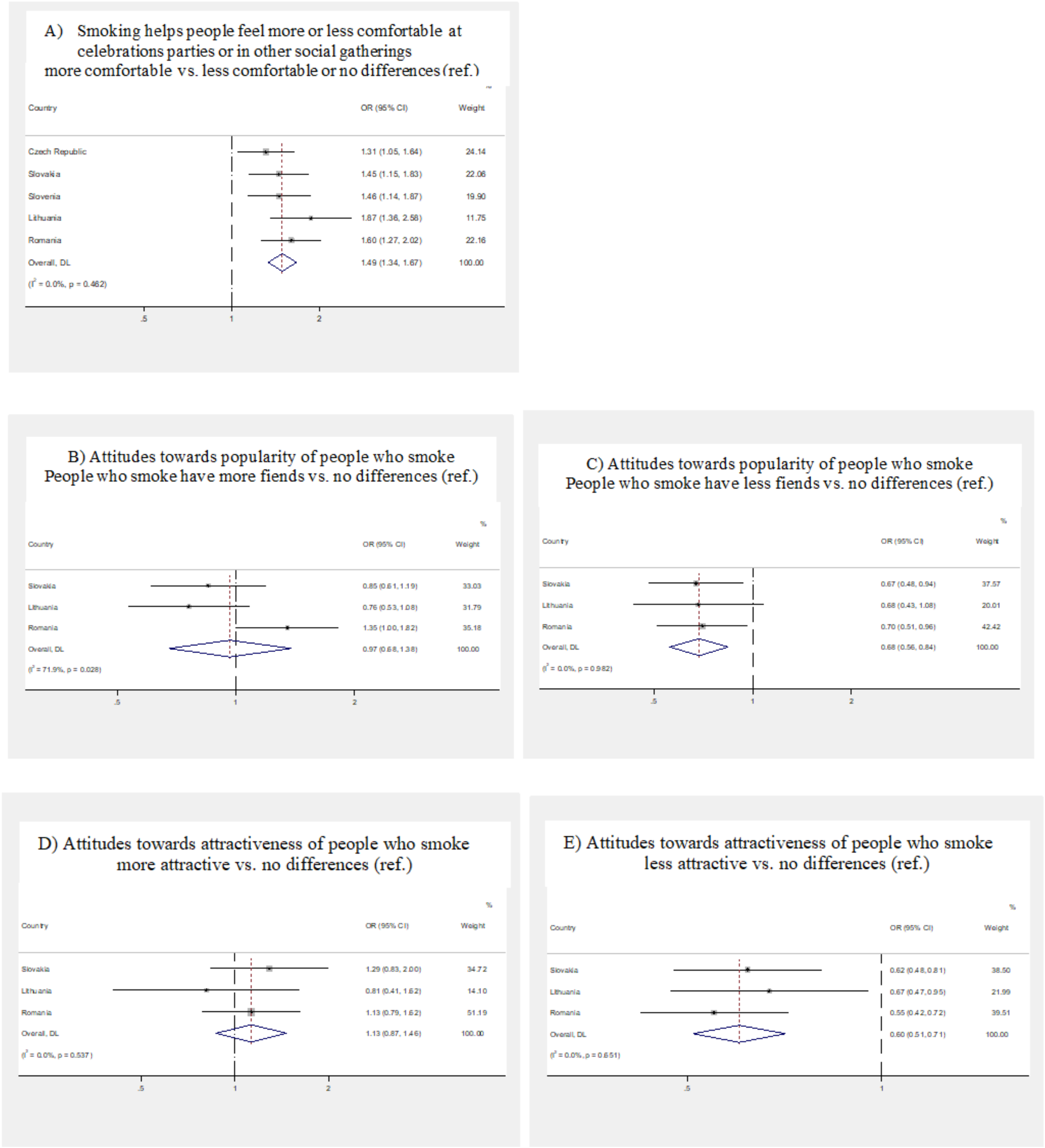

Fig. S5. Forest plots showing adjusted associations of attractiveness and popularity of smoking and susceptibility to tobacco use among never smoking youth from five central and eastern European countries. Country-specific odds ratio (OR) and 95% confidence interval (CI) were estimated by multivariable logistic regression models, and overall OR and 95% CI were estimated by random-effects meta-analysis by country. The following factors were included in the model: sex, age, money available for own expenses, SHS exposure at home and in public places, parental and peers smoking, knowledge about harmfulness of active and passive smoking, difficulty of quitting smoking, attractiveness and popularity of smoking, antismoking education provided by the school or family, tobacco advertising at point of sale, noticing people smoking at school and in movies. I^2, percentage of the total variability attributable to between-country heterogeneity; p, p value of heterogeneity using the Cochran's Q test.
